# IJCARS—IPCAI 2021 Special Issue: Information Processing for Computer-Assisted Interventions, 12th International Conference 2021—Part 1

**DOI:** 10.1007/s11548-021-02401-5

**Published:** 2021-05-15

**Authors:** Alfred Franz, Orcun Goksel

**Affiliations:** 1 Institute for Computer Science, Ulm University of Applied Sciences (THU), Ulm, Germany; 2 Department of Computer Assisted Medical Interventions, German Cancer Research Center (DKFZ), Heidelberg, Germany; 3 Department of Information Technology and Electrical Engineering, ETH Zurich, Zurich, Switzerland; 4 Department of Information Technology, Uppsala University, Uppsala, Sweden

It is a great pleasure to present this special issue of IJCARS, as Part 1 of the proceedings for the 12th Annual International Conference on Information Processing for Computer-Assisted Interventions (IPCAI) 2021. Given the number of scientific papers and the review timelines, the IPCAI 2021 proceedings will span two IJCARS special issues, and Part 1 herein features 16 of the papers to be presented at this year's IPCAI. As in the previous years, IPCAI this year will be held in conjunction with the Computer Assisted Radiology and Surgery (CARS) Congress. The contributions will be presented in a mix of pre-recorded videos as well as live summaries and discussion sessions on June 22 and 23. The event will be in a virtual format due to health and travel restrictions of the ongoing COVID-19 pandemic.

Starting in 2010, the annual IPCAI meetings have been a leading forum for discussing latest developments in computer assisted interventions (CAI). With a strong emphasis on research and practice, IPCAI focuses on computer-based tools and methodologies to support medical interventions, with the overall objective of enabling precise and safe systems for CAI. The IPCAI community aims to develop and test novel tools for enhanced planning, real-time imaging, guidance, navigation, and visualization, as well as situation awareness, cognition, and training. To promote translational research, the IPCAI meetings seek to showcase scientific work presenting novel theory, technical solutions, clinical applications, and hardware and software systems, as well as their validation. The key topics presented and discussed at IPCAI include (1) image processing and instrumentation for CAI; (2) interventional imaging; (3) surgical data science; (4) robotics; (5) tracking and navigation; (6) planning and simulation; (7) visualization; and (8) system evaluation and validation.

What distinguishes IPCAI from several other conferences is that the submitted manuscripts are expected to be of journal quality, with the goal to publish all accepted manuscripts, as in the case of this special issue of IJCARS. Accordingly, a rigorous review process is followed, through which the authors revise and improve their papers similarly to typical journal review processes. Each IPCAI submission has been reviewed by at least three external reviewers, and the final decisions are taken with the feedback and the recommendation of two independent Area Chairs. For any borderline cases, the involvement of and feedback from the IPCAI Board are sought by the Program Chairs. If and when further revisions are deemed necessary, these have been carried out via additional reviewing rounds, further led by the editorial team of the IJCARS, so that the manuscripts meet the stringent quality for journal publication.

IPCAI 2021 received 50 full-length manuscript submissions to be considered as Regular Papers. These were notably high-quality submissions, despite the severe impediments one can imagine that the research community has suffered due to the pandemic within the last year. The submissions encompass a wide geographic span, including in order of representation: Europe, North America, Asia, South America, and Africa. After two rounds of reviews, 33 of the submissions have been selected for presentation at IPCAI 2021, which will involve pre-recorded presentation videos as well as in-session summary and discussion time. Of these, 16 Regular Papers are included in this special issue Part 1. Other papers will later follow in a separate IJCARS special issue as IPCAI 2021 Part 2.

Following the positive reception of Long Abstract presentations in recent IPCAIs, we have followed this tradition and invited the community to submit Long Abstracts. Alternative to full-length Regular Papers, Long Abstract submissions with a shorter content aimed at showcasing novel ideas, software platforms, or recent breakthroughs, including those with preliminary but limited experimental validation. In an accelerated peer-review process, each Long Abstract submission has been reviewed by at least two external reviewers. In the Long Abstract category, a total of 13 submissions were received, of which eight have been accepted for presentation. These will also be presented with pre-recorded videos and with discussion time slots during the online IPCAI event. For the first time this year, the accepted Long Abstracts have been also invited by IJCARS to be considered for publication as a short communication, pending further review.

The trend from the last years of IPCAI continues as machine learning methods are shown at an ever increasing rate to be successfully utilized in CAI. The papers in this special issue demonstrate endoscopic and open surgical interventions, and processing of imaging data from ultrasound, mobile C-arms, and X-ray computed tomography. Methods for improving the tracking of medical instruments using electromagnetic sensors and optical cameras are also presented herein. In addition, methods for the planning of neurological and prostate biopsy interventions are demonstrated. Surgical robotics and biomechanical simulation are other topics that are studied in the papers included in this special issue.

We would like to thank the Area Chairs and all the reviewers very much for their time investment and dedication. Their help in selecting outstanding manuscripts and their feedback in the improvement of these through the revisions were invaluable in raising the scientific value of these contributions and maintaining the high quality of IPCAI. These will also no doubt help stimulate exciting discussions at the meeting.

We further acknowledge our sponsors for their continued support over the years, which has enabled us to recognize outstanding scientific work with reputable awards. We also thank all of the CARS Congress Organizers and the IJCARS Editorial Office for their continued help and guidance.

Last but not the least, we are very grateful to all the authors for choosing IPCAI to submit their valuable contributions and hope that the readers enjoy this special issue. The success of IPCAI is only possible with your ongoing support.

